# Combined polysaccharides from *Angelica sinensis*, *Crataegus pinnatifida*, *Prunus persica*, and *Carthamus tinctorius* attenuate cold exposure-induced bone loss by modulating the gut microbiota and fecal metabolites

**DOI:** 10.3389/fmicb.2026.1768890

**Published:** 2026-03-27

**Authors:** Lu Jin, Xiangyu Bian, Guanyu Zhang, Jinyu Zhu, Xi Li, Danfeng Yang

**Affiliations:** Military Medical Sciences Academy, Academy of Military Science, Tianjin, China

**Keywords:** bone loss, cold exposure, fecal metabolites, gut microbiota, polysaccharide

## Abstract

**Background:**

Chronic cold stress is a significant risk factor for skeletal deterioration; however, effective therapeutic strategies targeting the underlying environmental-metabolic interactions remain unclear. This study investigated the osteoprotective potential of Mixed Polysaccharides (MPs) and elucidated the mediating role of the gut microbiome.

**Methods:**

Cold exposure-induced bone loss was established in rats. Fecal microbiota transplantation (FMT), 16S rRNA gene sequencing, and untargeted metabolomics was employed to illustrate the positive effect of MPs on the improvement of cold-exposed bone loss.

**Results:**

MPs treatment effectively reversed cold-induced trabecular microarchitecture deterioration and bone mass loss. In femoral tissue, MPs rebalanced skeletal turnover by upregulating osteogenic markers (Runx2, Osterix) and suppressing osteoclastogenic factors (TRAP, c-fos), concurrent with a marked reduction in the levels of pro-inflammatory cytokines TNF-α and IL-1β in femur. Furthermore, MPs restored intestinal barrier integrity by upregulating tight junction proteins (ZO-1, Occludin), thereby mitigating the intestinal barrier impairment driven by cold stress. FMT experiments demonstrated that the osteoprotective effects of MPs are microbiota-dependent, as the transplantation of MPs-modulated microbiota recapitulated the bone-preserving and barrier-restoring phenotypes in recipient mice. Multi-omics integration identified that MPs selectively promoted the expansion of *Lactobacillus intestinalis* and the accumulation of cholylhistidine. Correlation analysis further revealed a strong link between the enrichment of these microbial and metabolic signatures, reduced pro-inflammatory cytokine levels, and improved bone formation.

**Conclusion:**

Our findings indicate that MPs alleviate cold-stress-induced bone loss by remodeling the gut microbiota and metabolic profile, fortifying the intestinal barrier and decreasing pro-inflammatory cytokine.

## 1 Introduction

Chronic cold exposure is a significant environmental stressor that disrupts energy homeostasis and nutrient metabolism, precipitating systemic metabolic disturbances (Briganti et al., 2023; Xue et al., 2025). Among these pathological consequences, skeletal deterioration is particularly notable. Epidemiological evidence indicates that populations residing in cold environments exhibit reduced cortical bone thickness, lower bone mineral density (BMD), and accelerated age-related bone loss (Wallace et al., 2014; Manolagas et al., 2013; van der Velde et al., 2017). Experimental studies further corroborate these findings, demonstrating that rats exposed to 4 °C for 14 days suffer from significant reductions in bone volume fraction and overall bone density (Serrat et al., 2008). These observations collectively underscore the deleterious impact of cold stress on skeletal integrity; however, the underlying mechanisms remain to be fully elucidated.

Bone remodeling relies on the dynamic equilibrium between osteoblastic bone formation and osteoclastic resorption. This balance is often disrupted by inflammation or metabolic stress, leading to bone loss, microarchitectural deterioration, and an increased fracture risk (Kyle et al., 2011). Emerging evidence suggests that the “gut-bone axis” plays a pivotal role in this process. The gut microbiota modulates bone metabolism through immune regulation, hormonal secretion, and production of metabolites such as short-chain fatty acids (SCFAs) (Liu et al., 2023; Wang et al., 2021; Coburn et al., 2013). Crucially, cold exposure has been shown to induce intestinal dysbiosis, characterized by a decline in beneficial SCFA-producing bacteria (e.g., *Roseburia*, *Bifidobacterium*) and an expansion of pathogenic taxa (e.g., *Bacteroides*, *Ruminococcaceae*) (Ma et al., 2020; Li C. et al., 2019; Rettedal et al., 2021; Wei et al., 2021; Ling et al., 2021). This microbial imbalance compromises the intestinal barrier and triggers systemic inflammation, which in turn accelerates osteoclastic activity and bone loss.

Current pharmacological interventions for bone loss, primarily bisphosphonates, are effective but limited by potential adverse effects, necessitating the development of safer alternatives (Deardorff et al., 2022). Active polysaccharides, as a kind of biological macromolecule derived from plants, have prebiotic functions in regulating the colonic microbiota (Ho Do et al., 2021; Fang et al., 2019). Therefore, plant polysaccharides targeting the intestinal flora have the potential to improve bone metabolic disorders. Studies have shown that *Achyranthes bidentata* polysaccharide can restore the bone micro-architecture (Zhang et al., 2018), *Polygonatum sibiricum* polysaccharide reduced expression of bone resorption markers (Zeng et al., 2011). Polysaccharides derived from medicinal herbs, specifically *Angelica sinensis* (AS), *Crataegus pinnatifida* (CP), *Prunus persica* (PP), and *Carthamus tinctorius* (CT), present a promising therapeutic strategy.

Therefore, this aimed to investigated the synergistic effect of polysaccharides from *Angelica sinensis*, *Crataegus pinnatifida*, *Prunus persica*, and *Carthamus tinctorius* on chronic cold-induced bone loss. In addition, to clarify the role of the mixed polysaccharides in the bone metabolism of cold-exposed rats, we performed fecal microbiota transplantation (FMT) and correlation analysis between differential microbiota and fecal metabolites. The findings of this study will provide a theoretical basis for the effective intervention of cold exposure induced bone loss.

## 2 Materials and methods

### 2.1 Preparation of ASP, CPP, PPP, and CTP (mix polysaccharides, MPs)

Polysaccharides from AS, CP, PP, and CT were prepared by hot-water extraction followed by precipitation with 80% ethanol and deproteination by the Sevag reagent (Shi, 2016). The final polysaccharides were purified by dialysis using a dialysis membrane (MWCO = 3 kDa) and then lyophilization. The purity of extracted polysaccharides was assayed by phenol-sulfuric method and was shown in Table 1.

**Table 1:** The purity of the polysaccharide.

Polysaccharide	Purity of the polysaccharide (%)
*Angelica sinensis* polysaccharide (ASP)	98.43
*Crataegus pinnatifida* polysaccharide (CPP)	46.61
*Prunus persica* polysaccharide (PPP)	43.64
*Carthamus tinctorius* polysaccharide (CTP)	54.57

### 2.2 Animal treatment

In this study, female Sprague-Dawley rats, 8 weeks old and weighing 210 ± 10 g, were obtained from Beijing Vital River Laboratory Animal Technology Co., Ltd. (Beijing, China). All animal experiments were approved by the Ethics Committee of the Tianjin Institute of Environmental and Operational Medicine (IACUC of AMMS-04-2023-036), and were conducted in strict accordance with the Laboratory Animal Guideline for ethical review of animal welfare.

**Figure 1:**
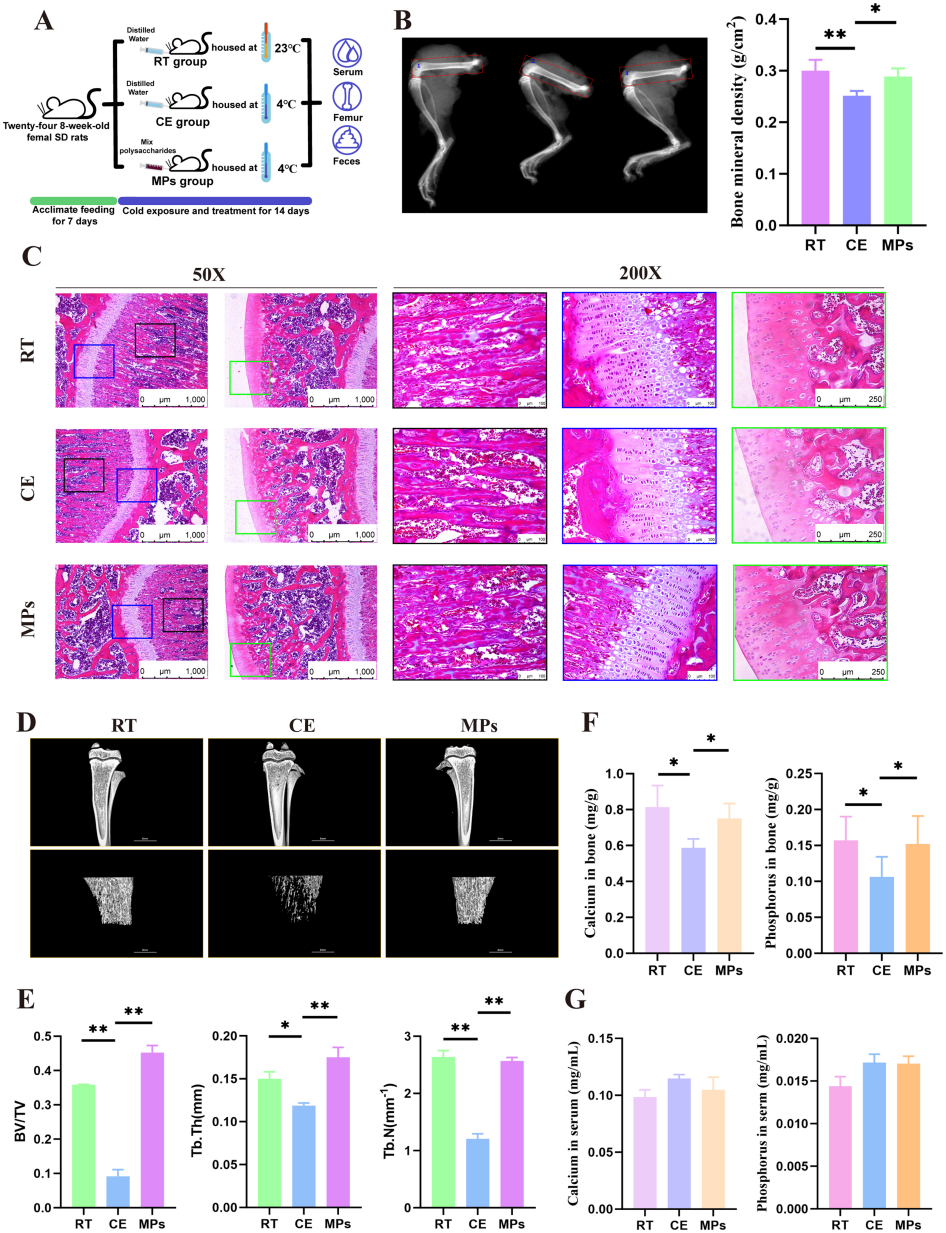
Effect of MPs intervention on bone damage in rats. **(A**) Flow chart of the MPs treatment experiment; **(B**) BMD of femur (*n* = 8); **(C**) HE-stained in femur (*n* = 5); **(D**) Micro CT of femur (*n* = 5); **(E**) BV/TV, Tb.Th and Tb.N of femur (*n* = 5); **(F**) bone calcium and phosphoruscontent of femur (*n* = 8); **(G**) serum calcium and phosphorus content (*n* = 8); RT, room temperature group; CE, cold exposure group; MPs, mix polysaccharides group. The data are presented as means and SD; *p < 0.05, **p < 0.01.

After 1 week of acclimation, the rats were randomly divided into three groups (*n* = 8): room temperature group (RT), cold-exposure group (CE), and cold-exposure supplemented with MPs group. The experimental procedure was shown in Figure 1A. Rats in the RT and CE groups were administered 2 mL of distilled water daily, while rats in the MPs group received 2 mL of a polysaccharide solution daily (AS polysaccharide 16.66 mg/kg, CP polysaccharide 16.66 mg/kg, PP polysaccharide 16.66 mg/kg, CT polysaccharide 16.66 mg/kg).

At the end of the experiment, rats were anesthetized with isoflurane. Blood was collected from the abdominal aorta. Femora, tibiae, and colon tissues were collected.

### 2.3 FMT

Rats were divided into three groups (*n* = 5 per group): T-RT, T-CE, and T-MPs. Fecal suspension was prepared as described in a previous study (Wei et al., 2024; Zhang et al., 2023). Briefly, stools from donor rats in each group were pooled, and 200 mg of stool was resuspended in 1 mL of sterile water. The suspension was vigorously mixed using a vortex for 10 s, followed by centrifugation at 800 g for 3 min. Each group (T-RT, T-CE, and T-MPs) was received the fecal suspension from the corresponding RT, CE, and MPs groups. Oral gavage was performed every 2 days for 14 days, with each rat receiving 10 mL/kg/day of the fecal suspension. All procedures were carried out under aseptic conditions.

### 2.4 Bone morphology, bone mineral density (BMD) analysis

The microstructure of left femur was analyzed using micro-computed tomography (micro-CT) (NEMO II Micro CT; PingSheng, China). The BMD of femoral tissue was measured using peripheral dual-energy X-ray absorptiometry (pDEXA, Norland/Stratec, Stratec Medizintechnik, Pforzheim, Germany). Ultra-high-resolution scanning mode was applied using Norland software.

### 2.5 Histological examination and immunohistochemistry

Based on previous studies, rat distal colon tissues and left femurs were collected, fixed in 4% paraformaldehyde solution, embedded in paraffin, and sectioned into 5 μm slices for subsequent hematoxylin and eosin (H&E) staining. Pathological assessment was performed following the method outlined in a prior study (Wen-Yin et al., 2024).

Immunohistochemistry was conducted using the procedure described by Qu et al. (2021). More than three random visual fields were selected for observation under a microscope. Quantitative analysis of the target proteins was performed using ImageJ software (version 1.5.7, National Institutes of Health, USA).

### 2.6 Measurement of biochemical parameters

Bone alkaline phosphatase (BALP), osteocalcin (BGP), pro-collagen I C-terminal propeptide (PICP), propeptide of type I procollagen (PINP), tartrate-resistant acid phosphatase (TRAP), C-terminal peptide of type I collagen (CTX-I), Lipopolysaccharide (LPS), Diamine Oxidase (DAO), and D-Lactic Acid (D-LA) in serum were measured using ELISA kits from Jianglai Biological (Shanghai, China). TNF-α, IL-1β, IL-10 in femur were measured using ELISA kits from Jianglai Biological (Shanghai, China). Serum phosphorus and ionized calcium levels were determined using a commercial kit from Nanjing Jiancheng Bioengineering Institute Co., Ltd. (Nanjing, China). Calcium and phosphorus content in bone tissues was measured using an atomic absorption spectrometer from Beyotime Biotechnology (Beijing, China). All procedures were performed according to the manufacturer’s instructions.

### 2.7 Western blot

The expression levels of tight junction (TJ) proteins in the colon and osteogenesis-specific transcription factors in the femur were analyzed by Western blotting, following the methodology described in our previous study (Bian et al., 2023). Total protein extracts from colon tissues were prepared using ice-cold radioimmunoprecipitation assay (RIPA) lysis buffer. The extracted proteins were denatured by boiling at 95 °C for 5 min in sample loading buffer. Equal amounts of proteins (30 μg) were loaded onto sodium dodecyl sulfate-polyacrylamide gel electrophoresis (SDS-PAGE) gels for separation, followed by transfer to a polyvinylidene fluoride (PVDF) membrane. After blocking with 5% skim milk in TBST buffer for 2 h at room temperature, the membranes were incubated with primary antibodies, followed by secondary antibody incubation. Blotting signals were detected using enhanced chemiluminescence (Merck Millipore, USA) and recorded on Dual energy X-ray absorptiometry (Norland, USA). Blot analysis was performed using Image Pro Plus 6.0 software, and band intensities were quantified using ImageJ. The antibodies used in this experiment were as follows: Col1a1 antibody (Bioworld, Nanjing, China), Osterix antibody (Abcam, MA, USA), RUNX2 antibody (Bioworld, Nanjing, China), OPN antibody (Abcam, MA, USA), TRAP antibody (Abcam, MA, USA), c-jun antibody (Abcam, MA, USA), c-fos antibody (Abcam, MA, USA), ZO-1 antibody (Abcam, MA, USA), Occludin (Bioworld, Nanjing, China), Claudin-1 (Abcam, MA, USA), GAPDH antibody (Bioworld, Nanjing, China), and anti-rabbit IgG, HRP-linked antibody (Cell Signaling Technology, MA, USA).

### 2.8 16S rRNA analysis

Colonic contents were collected from the rats, and microbial DNA was isolated. The concentration and purity of the DNA were assessed. Genomic DNA was extracted from the colonic contents using the TIANamp Soil DNA Kit (Tiangen Biotech, China), and the total DNA was further purified using a DNA purification kit (Tiangen DNA Gel Extraction Kit, China).

The V3-V4 region of the 16S rRNA gene was amplified using primers F (5′-ACTCCTACGGGAGGCAGCA-3′) and R (5′-GGACTACHVGGGTWTCTAAT-3′). High-throughput sequencing was performed on an Illumina platform following PCR product purification. Feature sequences were classified using the Naive Bayes Classifier, with SILVA serving as the reference database. The data were filtered with the following criteria: a base quality score greater than 50% and mass greater than 20. The clean tags were clustered into operational taxonomic units (OTUs) with a similarity threshold of 97%. To assess microbial community composition and *α*-diversity, the RDP Classifier (v.2.2) and mothur (version 1.33.3) were used. α-diversity was primarily evaluated using indices such as the Shannon index, Simpson index, Chao1 index, ACE index, and PD-whole-tree index. The Linear Discriminant Analysis Effect Size (LEfSe) method was applied to identify species with differential abundance.

### 2.9 Untargeted metabolomics analysis

Chemical compounds in rat stool samples were analyzed using untargeted liquid chromatography-mass spectrometry (LC–MS) at BGI Genomics Co., Ltd. (Shenzhen, China). A 50 mg stool sample was added to 600 μL of L-2-Chlorophenylalanine in methanol and centrifuged at 12,000 rpm at 4 °C for 10 min. The supernatant was then filtered through a 0.22 μm membrane. Chromatography was performed using an ACQUITY UPLC^®^ HSS T3 column (150 × 2.1 mm, 1.8 μm) (Waters, Milford, MA, USA), with the column maintained at 40 °C. The flow rate and injection volume were set to 0.3 mL/min and 2 μL, respectively. Gradient conditions for the mobile phases were based on the method described by Zelena et al. (2009). Mass spectrometric detection was carried out on an Orbitrap Exploris 120 (Thermo Fisher Scientific, USA) with electrospray ionization (ESI-MS) in both positive and negative modes. The spray voltages were set to 3.5 kV and −2.5 kV, respectively. Sheath gas, auxiliary gas, and capillary temperature were set to 30 arbitrary units, 10 arbitrary units, and 325 °C, respectively. The mass spectrometer scanned in the m/z range of 81–1,000, with a mass resolution of 60,000. Raw LC-MS data were converted to mzXML format and processed using XCMS for peak detection, retention time alignment, and normalization. Noise filtering and batch effect correction were applied to improve data reliability. Metabolites were annotated by searching the BGI Metabolome Database. Raw peak area data were log-transformed, and instrumental drift was corrected using a regression model established from QC samples. The ComBat algorithm was used to adjust for batch effects arising from sample preparation.

Multivariate statistical analyses, including principal component analysis (PCoA) and orthogonal partial least squares-discriminant analysis (OPLS-DA), were conducted to identify metabolic differences between groups. Differential metabolites were selected based on a variable importance in projection (VIP) score > 1.0 and *p* < 0.05. Pathway enrichment analysis was performed using MetaboAnalyst to identify key metabolic pathways associated with the observed changes.

### 2.10 Statistical analysis

Data analysis was performed using IBM SPSS Statistics (version 25.0). All experimental data are presented as the mean ± standard deviation (SD). To assess differences between two groups, a *t*-test was used, while differences among more than two groups were evaluated using one-way analysis of variance (ANOVA). LSD test and Bonferroni *post-hoc* test were applied for pairwise comparisons to identify statistically significant differences. Statistical significance was defined as *p* < 0.05 and *p* < 0.01.

## 3 Results

### 3.1 MPs ameliorate cold exposure-induced bone loss in rats

After 14 days of cold exposure, BMD of rats was decreased significantly. Meanwhile, the results of HE staining of femur tissue also showed revealed marked trabecular fragmentation, alongside reduced trabecular thickness and number in cold-exposed rats. However, administration of MPs effectively mitigated these defects. Micro-CT analysis showed that, compared to the CE group, the MPs-treated group exhibited a significantly higher BMD (*p* < 0.01). Compared to the CE group, the bone volume fraction (BV/TV) and trabecular number (Tb.N) wer increased by 15 and 7% in MPs-treated rats, respectively (*p* < 0.05, Figures 1B–E). Furthermore, femoral calcium and phosphorus contents were also restored by MPs treatment (*p* < 0.05, Figures 1F,G), indicating that MPs promote bone mineralization and preserve trabecular architecture under cold exposure.

### 3.2 MPs enhance bone formation and suppress bone resorption in cold-exposed rats

**Figure 2:**
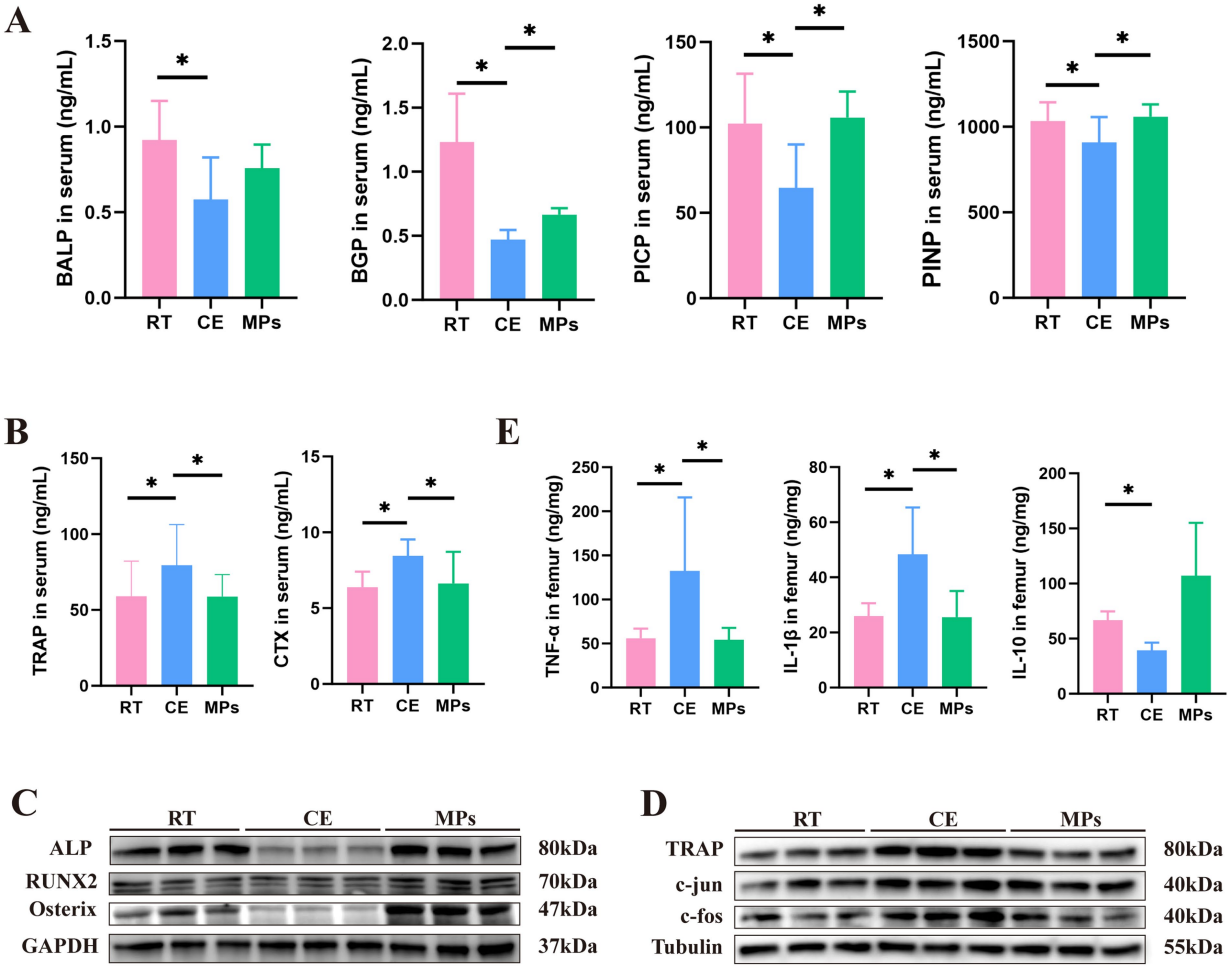
Effect of MPs intervention on bone resorption and bone formation. **(A)** BALP, BGP, PICP, and PINP content in the serum (*n* = 8); **(B)** TRAP and CTX-I content in the serum (*n* = 8); **(C)** Protein expression of osteogenic markers in the femur (*n* = 3); **(D)** Protein expression of bone resorption markers in the femur (*n* = 3); **(E)** TNF-α, IL-1β, and IL-10 content in the serum (*n* = 8); RT, room temperature group; CE, cold exposure group; MPs, mix polysaccharides group. The data are presented as means and SD; *p < 0.05.

To evaluate the systemic effects of MPs on bone turnover, we quantified serum markers of bone formation and resorption. Cold exposure significantly suppressed bone formation markers, including BALP, BGP, PICP, and PINP, compared to the RT group (*p* < 0.05, Figure 2A). Conversely, treatment with MPs markedly reduced the levels of bone resorption markers (TRAP and CTXI) relative to the CE group (*p* < 0.05, Figure 2B). We also assessed the expression of osteogenesis-specific transcription factors, we examined the expression of key osteogenic regulators in femoral tissue. While cold exposure downregulated the protein expression of Col1a1, ALP, Osterix, and Runx2, MPs supplementation effectively reversed this suppression (*p* < 0.05) (Figure 2C). These results suggest that MPs exert a dual protective effect by promoting bone formation and inhibiting resorption markers.

The c-fos/c-jun heterodimer acts as a critical transcription factor complex that translocate to the nucleus to drive the expression of TRAP, a key mark of osteoclast activity. We found that cold exposure significantly upregulated the expression of c-fos, c-jun, and TRAP. However, MPs supplementation suppressed these elevations. These data demonstrate that MPs may have the effect on inhibiting cold-induced bone resorption (Figure 2D).

TNF-α and IL-1β are known to inhibit bone formation by disrupting bone matrix production, alkaline phosphatase activity, osteoblast differentiation, and mineralization of osteoblast nodules (Zhou et al., 2006). We found that MPs treatment significantly attenuated the cold-induced elevation of TNF-α and IL-1β levels (*p* < 0.05). Concurrently, MPs administration markedly upregulated the expression of IL-10, a key anti-inflammatory cytokine (Figure 2E).

### 3.3 MPs alleviate the intestinal pathological histological damage and barrier function impairment in rats caused by cold exposure

**Figure 3:**
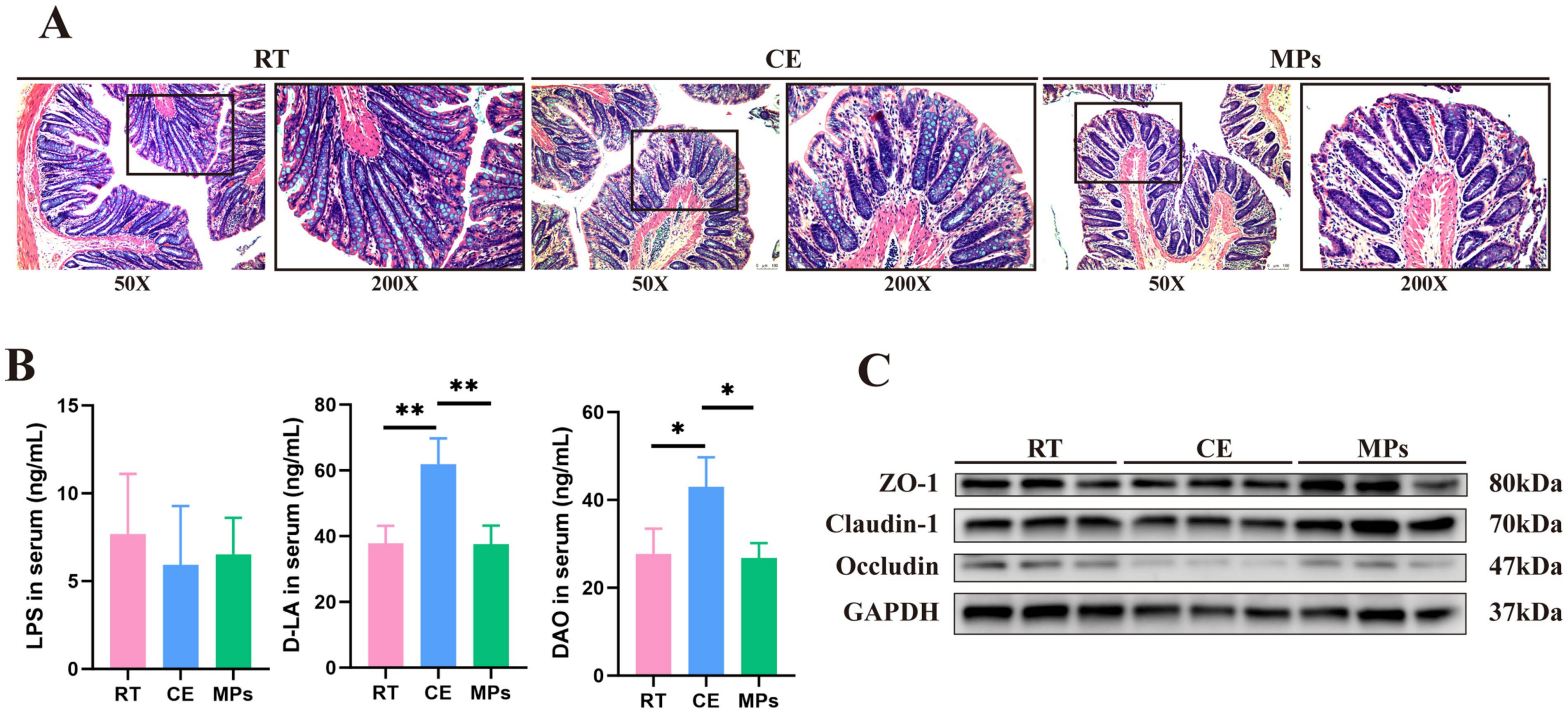
Effect of MPs treatment on the intestinal barrier structure. **(A)** Representative images of H&E-stained sections from each group; **(B)** LPS, DAO, and D-LA content in the serum (*n* = 8); **(C)** Protein expression of Claudin-1, Occludin, ZO-1, and GAPDH in colons (*n* = 3); RT, room temperature group; CE, cold exposure group; MPs, mix polysaccharides group. The data are presented as means and SD; *p < 0.05, **p < 0.01.

H&E staining revealed that cold exposure induced mucosal damage, characterized by crypt loss and a marked reduction in goblet cell density compared to the RT group. However, MPs intervention significantly ameliorated these changes, significantly restoring goblet cell numbers (Figure 3A).

To assess intestinal barrier function, we measured serum markers of permeability. The CE group exhibited elevated levels of DAO, and D-LA, indicating barrier dysfunction (*p* < 0.05). In contrast, MPs treatment significantly reduced these markers (Figure 3B).

Furthermore, we examined the expression of tight junction (TJ) proteins. MPs treatment upregulated the protein levels of ZO-1, Occludin, and Claudin-1 in the colon (Figure 3C). These findings suggest that MPs protect against cold-induced intestinal injury by preserving epithelial integrity and reinforcing the mucosal barrier.

### 3.4 MPs mitigate cold exposure-induced gut microbiota dysbiosis

**Figure 4:**
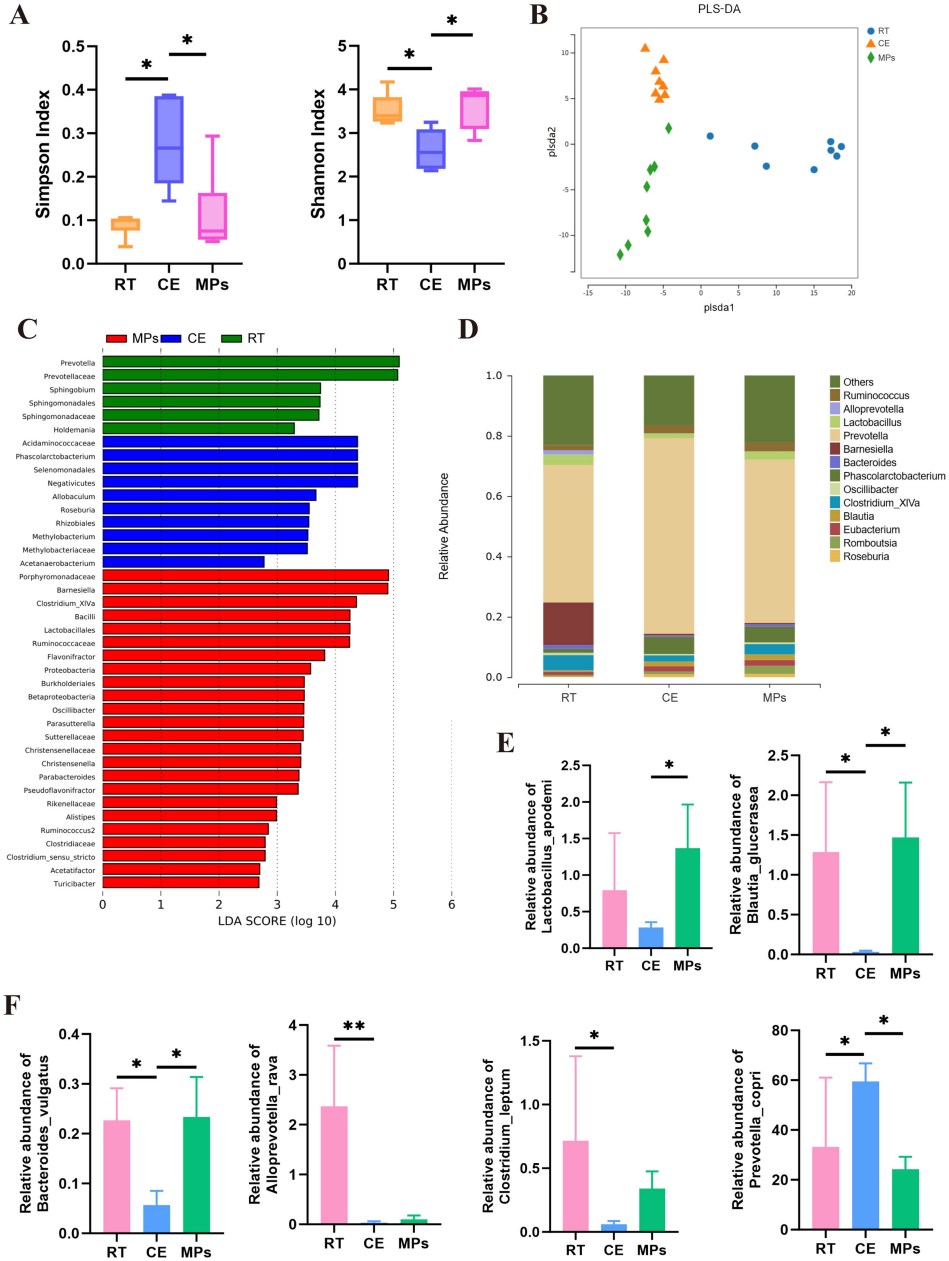
Effect of MPs treatment on the gut microbiota. **(A)** Shannon and Simpson index; **(B)** PLS-DA analysis results; **(C)** LEfSe analysis results; **(D)** Relative abundance of the gut microbiota composition among the three groups at the phylum level; **(E)** Relative abundances of Lactobacillus_apodemi, Blautia_glucerasea **(F)** Relative abundances of Bacteroides_vulgatus, Alloprevotella_rava, Clostridium_leptum, Prevotella_copri; RT, room temperature group; CE, cold exposure group; MPs, mix polysaccharides group. The data are presented as means and SD; *p < 0.05, **p < 0.01.

To investigate whether MPs could restore gut microbial homeostasis, we performed 16S rRNA sequencing. Alpha diversity analysis revealed that cold exposure significantly reduced microbial richness and evenness, as evidenced by a decreased Shannon index and increased Simpson index compared to the RT group. However, MPs treatment effectively reversed these changes (Figure 4A). PLS-DA analysis further demonstrated distinct clustering of microbial communities among the groups, indicating that MPs intervention shifted the overall microbiota composition away from the CE pattern (Figure 4B). To further investigate the effects of MPs on microbiota composition, we analyzed the data at both the phylum and species levels. At the phylum level, the abundances of *Prevotella* and *Clostridium_XlVa* were lower in both the RT and MPs treatment groups compared to the CE group, while the abundance of *Lactobacillus* was higher in the RT and MPs groups than in the CE group (*p* < 0.05, Figure 4D). LEfSe analysis (LDA score > 2.0) identified *Lactobacillus* as a key biomarker enriched in the MPs-treated group (Figure 4C), a finding consistent with species-level profiling (Figures 4E,F). These data suggest that MPs alleviate cold-induced dysbiosis, potentially by promoting the expansion of *Lactobacillus*.

### 3.5 The protective effect of MPs against bone loss is transferable via gut microbiota

**Figure 5:**
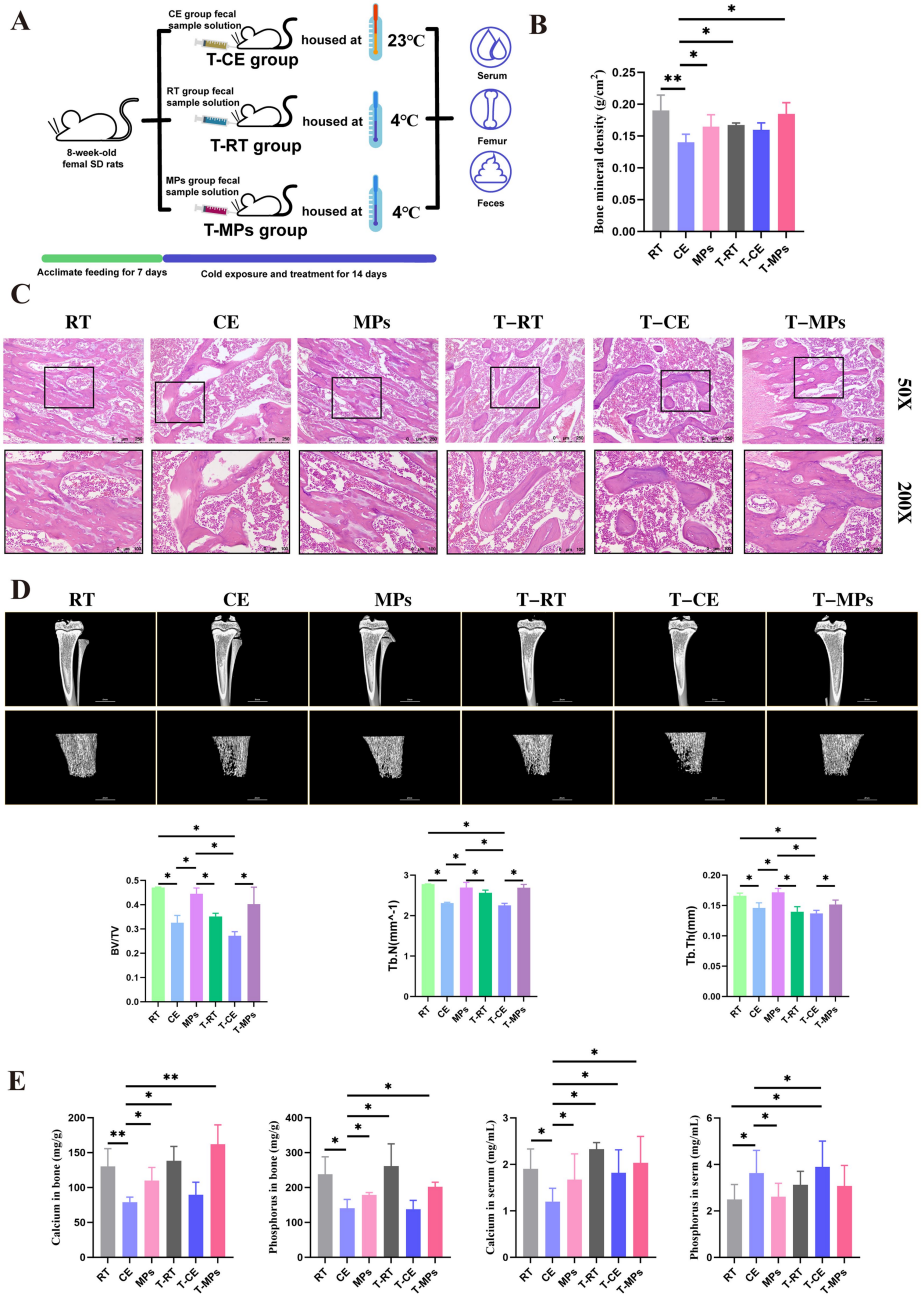
Effect of fecal microbiota transplantation after MPs intervention in cold exposure-induced bone loss rats. **(A)** Flow chart of the FMT experiment; **(B)** BMD of femur (*n* = 5); **(C)** HE-stained in femur (*n* = 3); **(D)** Micro CT, BV/TV, Tb.Th, and Tb.N of femur (*n* = 3); **(E)** Bone calcium and phosphorus content of femur, serum calcium and phosphorus content (*n* = 5); RT, room temperature group; CE, cold exposure group; MPs, mix polysaccharides group; T-RT, trans-room temperature fecal group; T-CE, trans-cold exposure fecal group; T-MPs, trans-mix polysaccharides fecal group. The data are presented as means and SD; *p < 0.05, **p < 0.01.

**Figure 6:**
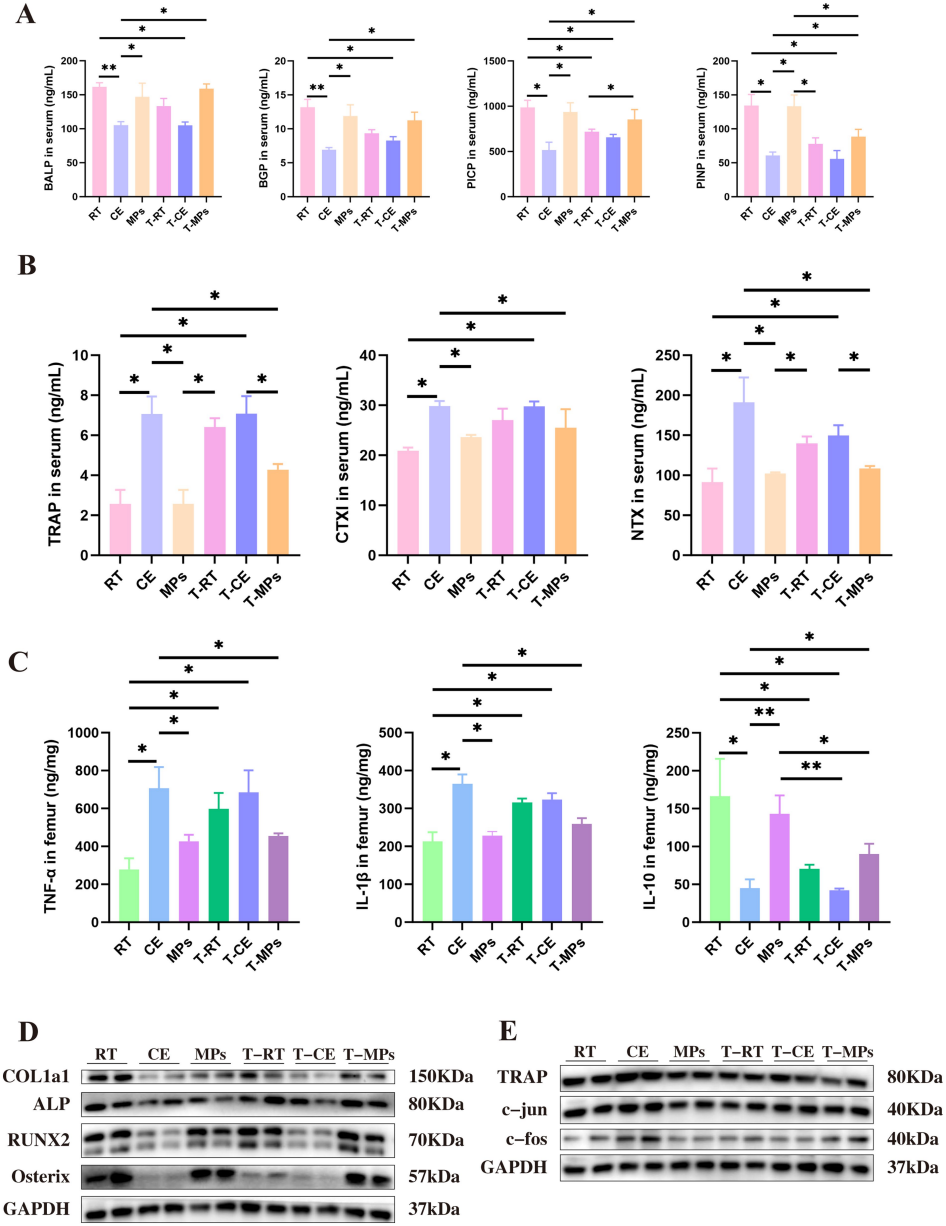
Effect of fecal microbiota transplantation after MPs intervention in bone resorption and bone formation. **(A)** BALP, BGP, PICP, and PINP content in the serum (*n* = 5); **(B)** TRAP, CTX-I, and NTX content in the serum (*n* = 5); **(C)** Protein expression of osteogenic markers in the femur (*n* = 3); **(D)** Protein expression of bone resorption markers in the femur (*n* = 3); **(E)** TNF-α, IL-1β, and IL-10 content in the serum (*n* = 5); RT, room temperature group; CE, cold exposure group; MPs, mix polysaccharides group; T-RT, trans-room temperature fecal group; T-CE, trans-cold exposure fecal group; T-MPs, trans-mix polysaccharides fecal group. The data are presented as means and SD; *p < 0.05, **p < 0.01.

To determine whether the intestinal microbiota mediates the osteoprotective effects of MPs, we performed FMT. Microbiota suspensions from the RT, CE, and MPs groups were administered to the T-RT, T-CE, and T-MPs groups, respectively (Figure 5A). Our study revealed that transplantation of microbiota from MPs-treated donors significantly attenuated cold-induced bone loss. Histologically, T-MPs mice displayed reduced femoral pathology and preserved trabecular architecture, characterized by significantly higher BV/TV, Tb.N, and BMD compared to the CE group (*p* < 0.05, Figures 5B,C and D). Consistent with these structural improvements, femoral calcium and phosphorus levels were markedly elevated (Figure 5E). Furthermore, FMT reversed the cold-induced imbalance in bone turnover, elevating serum bone formation markers (BALP, BGP, PICP, PINP) (*p* < 0.05, Figure 6A) while suppressing resorption markers (TRAP, CTXI) (Figure 6B). T-MPs mice exhibited upregulated expression of the osteogenic proteins Col1a1, ALP, Osterix, and Runx2, whereas the osteoclastogenic factors TRAP, c-jun, and c-fos, as well as inflammatory cytokines, were significantly downregulated in femoral tissue (Figures 6C–E). These data suggest that the MP-modulated microbiota is sufficient to mitigate cold exposure-induced bone deficits.

### 3.6 The ameliorating effect of MPs on intestinal barrier disruption is microbiota-dependent

**Figure 7:**
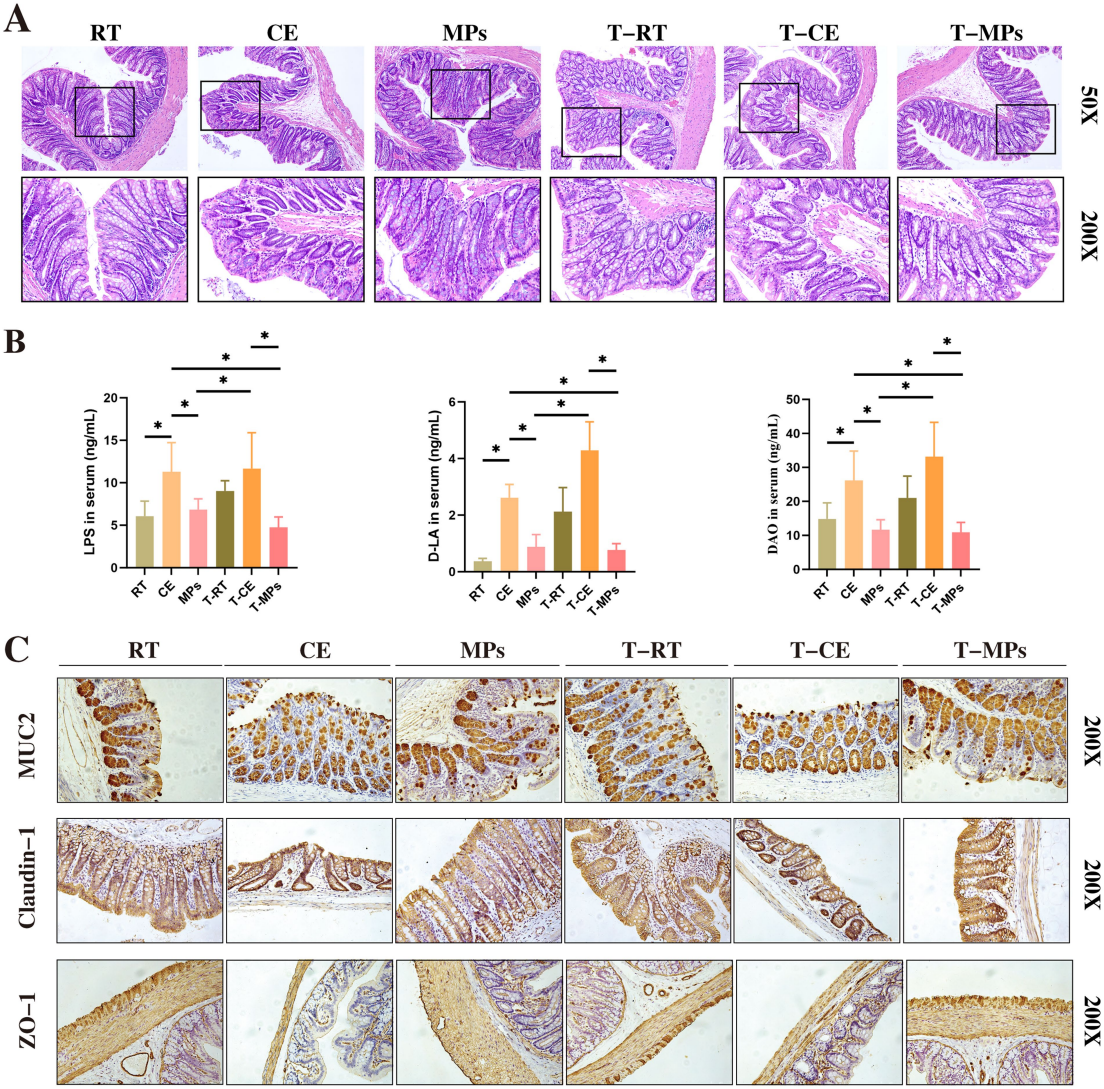
Effect of fecal microbiota transplantation after MPs intervention in the intestinal barrier structure. **(A)** Representative images of H&E-stained sections from each group; **(B)** LPS, DAO, and D-LA content in the serum (*n* = 5); **(C)** Immunohistochemical staining of Claudin-1, MUC2, and ZO-1 in colons (*n* = 3); RT, room temperature group; CE, cold exposure group; MPs, mix polysaccharides group; T-RT, trans-room temperature fecal group; T-CE, trans-cold exposure fecal group; T-MPs, trans-mix polysaccharides fecal group. The data are presented as means and SD; *p < 0.05.

**Figure 8:**
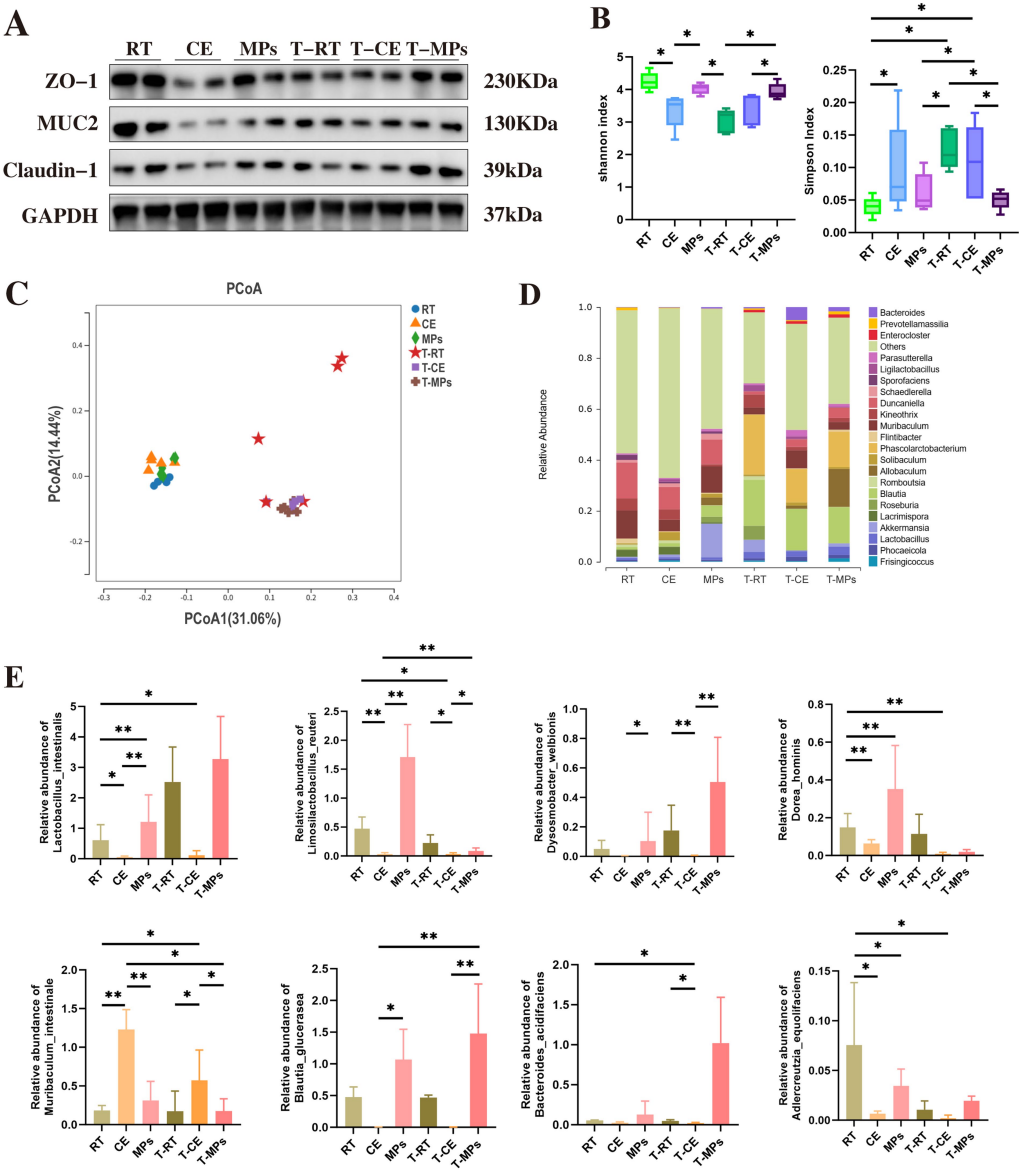
Effect of fecal microbiota transplantation after MPs intervention in the intestinal barrier structure and the gut microbiota. **(A)** Protein expression of Claudin-1, MUC2, ZO-1, and GAPDH in colons (*n* = 3); **(B)** Shannon and Simpson index; **(C)** PCoA analysis results; **(D)** Relative abundance of the gut microbiota composition among the six groups at the phylum level; **(E)** Relative abundances of Lactobacillus intestinalis, Limosilactobacillus reuteri, Dysosmobacter welbionis, Dorea hominis, Muribaculum intestinale, Blautia glucerasea, Bacteroides acidifaciens, and Adlercreutzia equolifaciens; RT, room temperature group; CE, cold exposure group; MPs, mix polysaccharides group; T-RT, trans-room temperature fecal group; T-CE, trans-cold exposure fecal group; T-MPs, trans-mix polysaccharides fecal group. The data are presented as means and SD; *p < 0.05, **p < 0.01.

H&E staining results demonstrated that T-MPs intervention markedly ameliorated colonic mucosal injury (Figure 7A). This restoration of structural integrity was paralleled by improved barrier function, as evidenced by significant reductions in serum permeability markers (*p* < 0.05, Figure 7B). Furthermore, T-MPs treatment effectively rescued the expression of colonic TJ proteins (Figures 7C, 8A).

### 3.7 MPs remodel gut metabolomic profiles and attenuate bone loss in a gut microbiota-dependent manner

To determine whether the osteoprotective effects of the gut microbiome were associated with specific microbial community changes, we analyzed 16S rRNA gene sequences from the CE and T-MPs groups. Alpha-diversity metrics indicated distinct community structures across treatments, the Simpson index was elevated in the CE and T-CE groups, whereas the Shannon index was significantly lower in the MPs and T-MPs groups relative to the RT controls (*p* < 0.05, Figure 8B). PCoA confirmed these shifts, showing distinct clustering of samples by treatment group (Figure 8C).

To further elucidate the effect of MPs-mediated transfer on the gut microbiota, we performed taxonomic analyses at both the genus and species levels. At the phylum level, we observed a marked expansion of Bacteroides and a concurrent depletion of *Lctobacillus* (Figure 8D). At the species level, several bacterial species were significantly increased in the T-MPs group compared to the CE group, including *Lactobacillus intestinalis*, *Limosilactobacillus reuteri*, *Blautia glucerasea*, and *Dorea hominis* (*p* < 0.05, Figure 8E).

**Figure 9:**
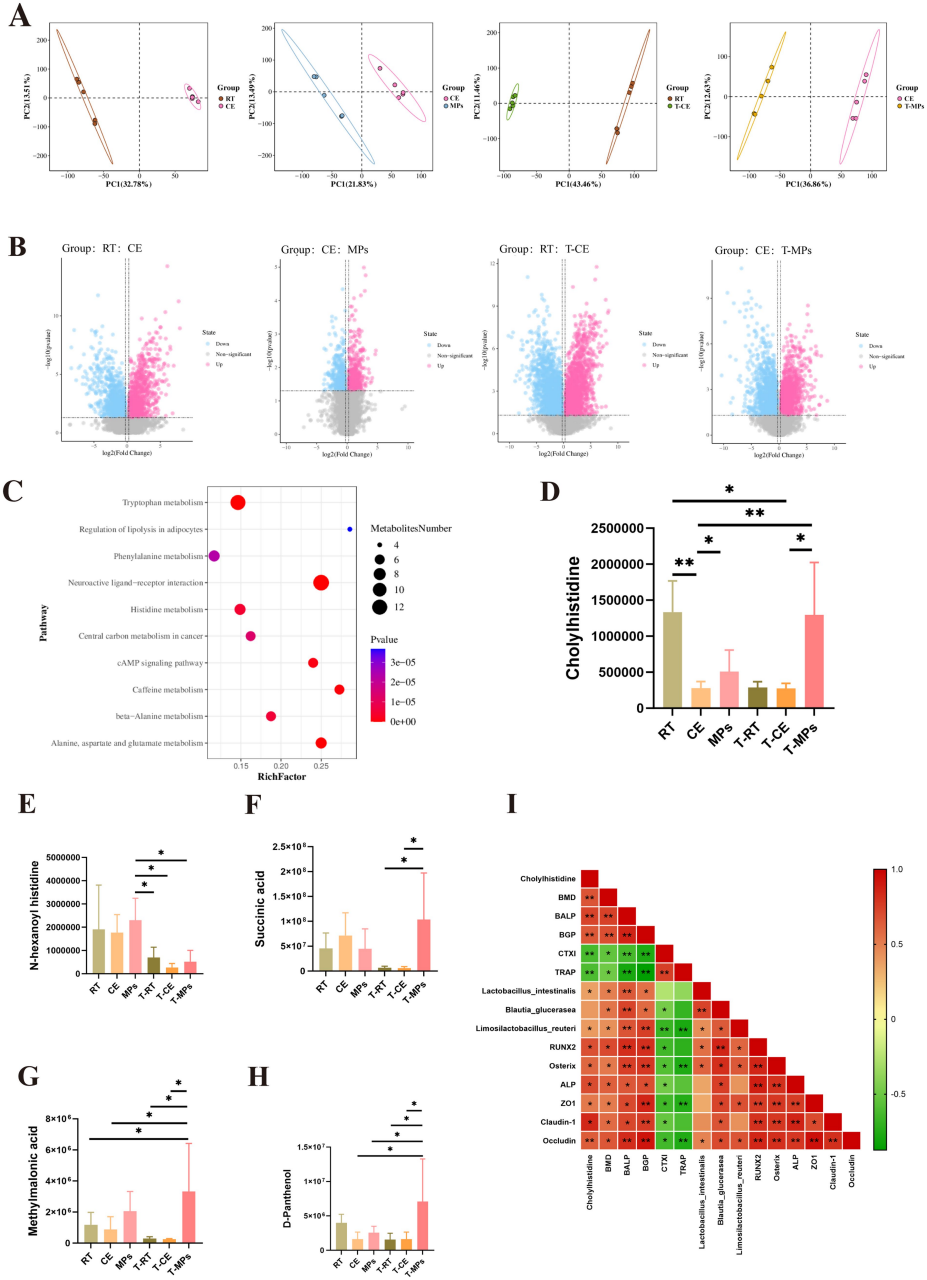
Effect of MPs intervention on intestinal metabolism. **(A)** PLS-DA result; **(B)** volcano result; **(C)** KEGG enrichment bubble diagram; **(D–H)** relative abundance of eight metabolites (Cholylhistdine, *N*-hexanoyl histidine, succinic acid, methylmalonic acid, and D-Panthenol); **(I)** Spearman’s correlation analysis; RT, room temperature group; CE, cold exposure group; MPs, mix polysaccharides group; T-RT, trans-room temperature fecal group; T-CE, trans-cold exposure fecal group; T-MPs, trans-mix polysaccharides fecal group. The data are presented as means ± SD; *p < 0.05, **p < 0.01.

To assess the functional impact of MPs on microbial metabolism, we performed untargeted fecal metabolomics. Partial least-squares discriminant analysis (PLS-DA) demonstrated robust separation across the RT, CE, MPs, and T-MPs groups (Figure 9A), with differentially abundant metabolites identified via volcano plots (Figure 9B). KEGG pathway enrichment analysis revealed that MPs intervention significantly upregulated arginine biosynthesis, as well as pathways involved in tryptophan, phenylalanine, and histidine metabolism (Figure 9C). Notably, MPs intervention significantly increased the level of specific metabolites, including cholylhistidine (Figure 9D), N-hexanoyl histidine (Figure 9E), succinic acid (Figure 9F), methylmalonic acid (Figure 9G), and D-panthenol (Figure 9H). Spearman correlation analysis further linked these metabolic shifts to host physiology: cholylhistidine levels correlated negatively with inflammatory markers and positively with BMD, osteogenic markers (BALP, RUNX2), gut barrier protein ZO-1, and *Lactobacillus intestinalis* (Figure 9I). These results indicate that MPs intervention remodeled intestinal metabolism, significantly enriched the arginine and histidine metabolic pathways, and increased the level of cholylhistidine.

## 4 Discussion

In the present study, MPs attenuate cold-induced bone loss by orchestrating a protective gut-bone axis. Specifically, MPs selectively promote the expansion of *Lactobacillus intestinalis* and the production of cholylhistidine, which in turn reinforce intestinal barrier integrity via the upregulation of tight junction proteins. This restoration of gut homeostasis dampens systemic inflammation, thereby suppressing resorption and enhancing formation-to preserve bone mass.

Dietary plant polysaccharides are established modulators of intestinal homeostasis (Wang et al., 2013). Consistent with this, we observed that MPs treatment selectively expanded the populations of beneficial commensals, notably *Lactobacillus intestinalis*, *Limosilactobacillus reuteri*, and *Blautia glucerasea*. This specific enrichment is likely driven by substrate availability, genomic analyses predict that *Limosilactobacillus reuteri* encodes the glycoside hydrolases and transferases necessary to metabolize plant-derived non-starch polysaccharides (Sun et al., 2025). *Limosilactobacillus intestinalis* and *Limosilactobacillus reuteri* are well-documented drivers of mucosal health, known to reinforce barrier integrity (Wang et al., 2023; Son et al., 2020). It is associate with the expression of MUC2, and inhibition of pro-inflammatory cytokines (TNF-α, IL-6) (Yang et al., 2025; Wang et al., 2020). Similarly, the depletion of *Blautia* species is a hallmark of inflammatory bowel disease (Chen et al., 2025). Collectively, our findings suggest that MPs fortify gut barrier function by fostering a synergistic guild of mucin-promoting and anti-inflammatory bacteria.

The intestinal barrier plays a crucial role in facilitating nutrient absorption while preventing the translocation of harmful substances across the intestinal epithelium (Schoultz and Keita, 2020). The integrity of this barrier relies on TJ complexes, composed of transmembrane proteins such as claudins, occludin, and ZO-1 (Otani and Furuse, 2020). Gut dysbiosis disrupts these structures and contributes to skeletal pathology. Previous study have shown that FMT from aged or osteoporotic rats into young recipients impairs intestinal barrier function, downregulates the expression of occludin, claudin, and ZO-1, and consequently trigger bone loss (Wang et al., 2022). These findings underscore the causal link between barrier dysfunction and bone metabolism. Notably, recent studies further demonstrate that environmental stressors such as cold exposure can disrupt gut microbial ecology and secondarily impair barrier function, thereby accelerating pathological bone remodeling (Indrio and Salatto, 2025; Lv et al., 2024). In the present study, cold exposure disrupted this delicate balance, as evidenced by the downregulation of ZO-1, occludin, and claudin-1, leading to increased intestinal permeability and elevated pro-inflammatory cytokines (IL-1β, TNF-α). Notably, MPs mitigated mucosal structural damage and significantly upregulated TJ protein expression. This barrier reinforcement coincides with the selective expansion of *Lactobacillus intestinalis* and the enrichment of cholylsarcosine. *Lactobacillus intestinalis* is a known enhancer of mucosal barrier function (Chen et al., 2023),and bile acid derivatives like cholylsarcosine have been reported to suppress intestinal inflammation (Schepper et al., 2017; Schepper et al., 2020; Ji et al., 2025). Our findings suggest that MPs may improve cold exposure induced bone loss by rectifying dysbiosis-induced barrier leakiness.

Pro-inflammatory cytokines are critical mediators of pathological bone remodeling (Klein, 2018; Griffin et al., 2015). It is well established that TNF-α and IL-1β disrupt skeletal homeostasis by potently stimulating osteoclast-mediated bone resorption while simultaneously inhibiting osteoblast-driven bone formation (Graves et al., 2011; Xu et al., 2023; Pacifici, 1996). TNF-α has been shown to synergize with RANKL to accelerate osteoclast differentiation (Lam et al., 2000; Komine et al., 2001). IL-1β and other inflammatory mediators amplify these RANKL-dependent catabolic signals, sustaining a microenvironment that favors progressive bone erosion. Therefore, targeting the production or downstream signaling of these cytokines represents a rational therapeutic strategy to arrest cold exposure-induced bone loss. Our data demonstrate that MPs treatment significantly upregulated the serum bone-formation markers BALP and PICP, as well as the femoral expression of osteogenic genes Runx2 and OPN, thereby counteracting the cold-induced suppression of osteogenesis. Conversely, MPs substantially reduced serum concentrations of the bone-resorption markers CTX-I and NTX, along with femoral TRAP activity. Importantly, these anabolic and anti-catabolic effects were accompanied by a marked reduction in femoral TNF-α and IL-1β levels. Collectively, these findings provide robust mechanistic evidence that MPs ameliorate cold exposure-induced bone loss by resolving local inflammation, thereby shifting the balance of bone remodeling towards formation and away from resorption.

Microbial metabolites serve as critical signaling mediators in host-microbiota cross-talk (Qi et al., 2022). In this study, metabolomic profiling revealed that MPs treatment profoundly reshaped the gut metabolic landscape, specifically enriching pathways involved in tryptophan, phenylalanine, histidine, and alanine/aspartate/glutamate metabolism. Of particular interest is the marked accumulation of cholylhistidine in the T-MPs group. Cholylhistidine functions as a potent agonist of the farnesoid X receptor (FXR), a nuclear receptor whose deficiency is causally linked to accelerated bone loss *in vivo* (Boufker et al., 2011; Xia et al., 2023). FXR activation is a well-established brake on osteoclastogenesis. FXR deficiency has been shown to disrupt the downstream JAK3-STAT1 signaling axis and alter IL-1β dynamics, thereby unleashing osteoclast formation (Li Z. et al., 2019). Our analysis highlighted a significant accumulation of cholylhistidine following T-MPs treatment. Given its correlation with improved skeletal parameters, this metabolite implies a potential metabolic link in the gut-bone axis that supports a net gain in bone mass.

Collectively, our findings demonstrate that MPs alleviate cold-induced bone loss by remodeling gut microbiota and fecal metabolic profiles. MPs preserve intestinal barrier integrity, likely via the enrichment of *Lactobacillus intestinalis* and accumulation of cholylhistidine. Although fecal microbiota transplantation experiments underscore the pivotal role of the gut microbiota, we cannot rule out contributions from non-microbial elements in the fecal suspension, including residual polysaccharides and host-derived metabolites, which may independently influence bone metabolism and barrier function. The absence of standard anti-osteoporotic agents as positive controls reflects our focus on elucidating the microbiota-dependent mechanisms underlying MPs’ osteoprotective effects; future investigations will incorporate such pharmacological benchmarks to assess therapeutic efficacy. Overall, this study identifies a novel mechanism by which MPs protect against cold-induced bone deterioration, supporting dietary polysaccharides as a promising strategy for bone health. Further studies will clarify the precise pathways by which *Lactobacillus intestinalis* and cholylhistidine regulate skeletal homeostasis under cold stress.

## Data Availability

The data presented in the study are deposited in the NCBI Sequence Read Archive (SRA) under the Bioproject number: PRJNA1392165.
